# Proactive risk stratification and low-risk penicillin allergy delabeling by an antimicrobial stewardship pharmacist

**DOI:** 10.1017/ash.2024.91

**Published:** 2024-05-27

**Authors:** Breana N. Caturano, Lauren L. Bjork, Paola N. Lichtenberger, Viviana M. Temiño

**Affiliations:** Miami Veterans Affairs Healthcare System, Miami, FL, USA

## Abstract

Penicillin (PCN) allergy delabeling is an important component of antimicrobial stewardship; however, widespread implementation has lagged. We found that most patients had low-risk PCN allergy histories eligible for delabeling without skin testing. Pharmacist-led risk stratification and drug challenge expanded access to delabeling independently from an Allergy/Immunology service.

## Introduction

Penicillin (PCN) remains the most reported drug allergy in the United States, with the prevalence ranging from 10% in the general population and up to 15% in hospitalized patients.^1^ Only 1%–10% of patients are found to have true PCN allergy when drug challenged.^1,2^ PCN allergy is associated with increased patient morbidity, colonization with multidrug-resistant bacteria, and higher healthcare costs.^1,2^ Despite the importance of PCN allergy delabeling for antimicrobial stewardship (ASP), widespread implementation in US healthcare facilities has lagged due to the limited size and variable availability of an allergy-trained workforce.^3–5^

The approach to PCN allergy delabeling has evolved over time with several methods in use today, including delabeling based on history alone, PCN skin testing (PST) with or without drug challenge (DC), and DC alone.^2^ Risk stratification assists with determining which method is most appropriate, and assessment tools such as the recently validated PEN-FAST aim at streamlining this process.^1,6^ Commonly accepted low-risk histories include isolated symptoms consistent with side effect, distant reaction (>10 years), unknown reaction, pruritus without rash, and patients with only a family history of PCN allergy.^2^ Up to 20% of adults with self-reported PCN allergy are non-immune reactions such as side effect and may be delabeled based on history alone.^2^ An estimated 50%–76% of adults with self-reported PCN allergy have low-risk histories and may be eligible for allergy clearance via DC alone.^6,8,9^ These findings may present an opportunity for developing delabeling programmes in hospitals where there is limited or no allergist presence, since DC does not require the specialized training, resources, and experience required for PST. We report the 8-month real-world experience of a low-risk PCN allergy delabeling inpatient programme managed by an ASP pharmacist without Allergy/Immunology oversight.

## Methods

### Clinical pharmacist scope of practice

The standard operating procedure for Competency Evaluation for Pharmacists with Scopes of Practice required a minimum of 20 full workdays of supervised training. From October 2021 to April 2022, 36 patients were evaluated and six amoxicillin drug challenges (ADC) were performed by an ASP pharmacist under the supervision of an allergist. The additional privileges request was approved by the Miami VA Healthcare System Professional Standards Board to include obtaining a PCN allergy history, revision of allergies where clinically appropriate, and administration of ADC.

### Setting and protocol

**Figure 1.**
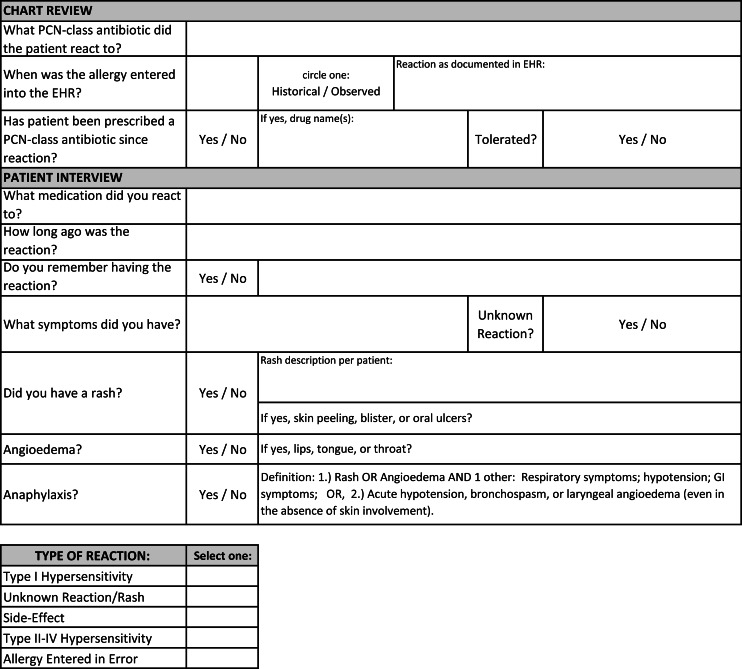
Penicillin allergy history. *PCN*, penicillin; *EHR,* electronic health record.

**Figure 2.**
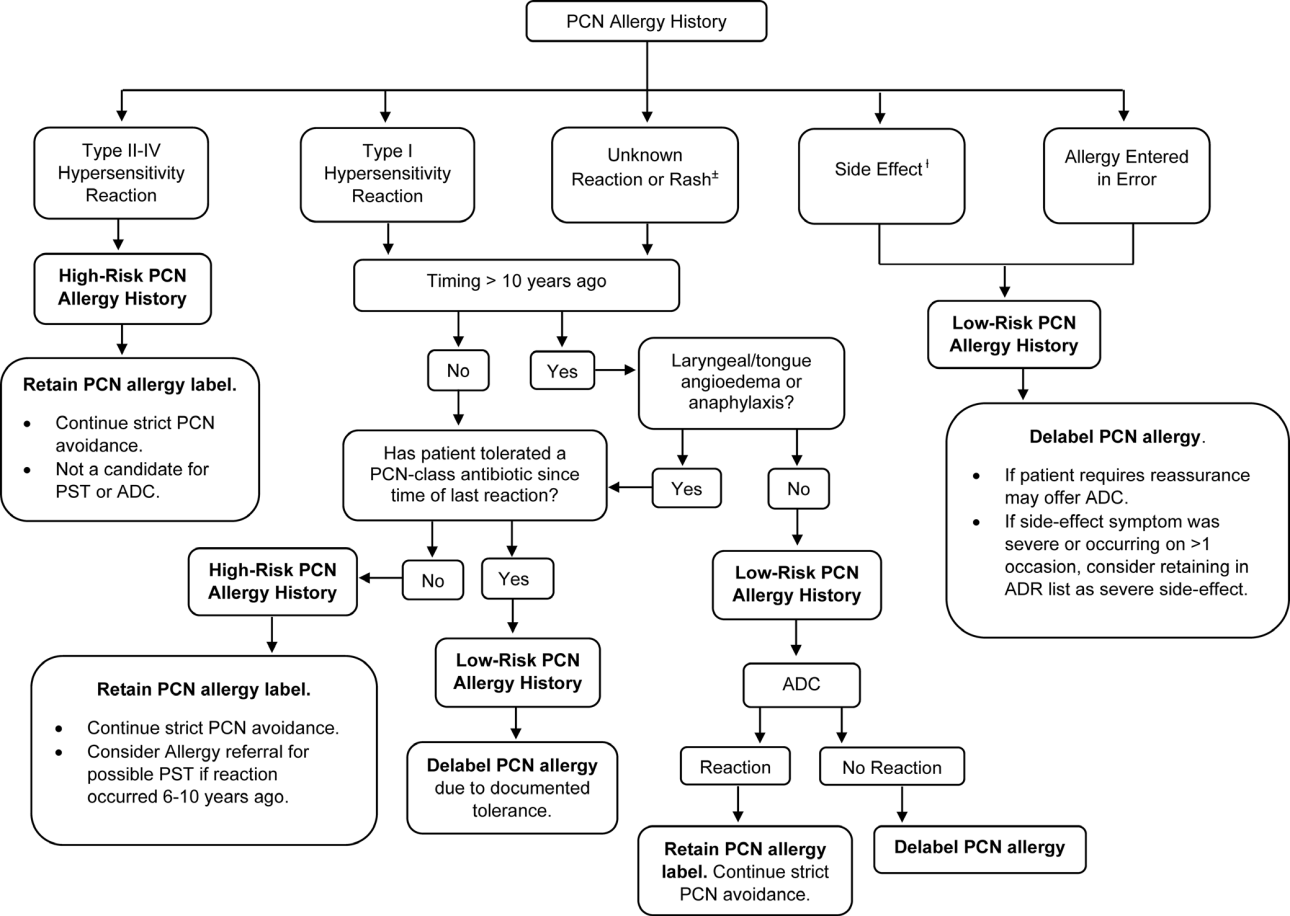
Penicillin allergy protocol. *PCN*, penicillin; *PST*, penicillin skin test; *ADC*, amoxicillin drug challenge. ^±^Rash: Benign cutaneous eruption that could not be clearly identified as urticarial but was not consistent with type 4 hypersensitivity such as severe cutaneous adverse reaction or acute generalized exanthematous pustulosis. ^ƚ^Side effect: Swelling at the site of injection, isolated gastrointestinal symptoms, subjective arthralgias and/or myalgias, headache, dizziness, altered mental status, vasovagal syncope, and seizure.

This was a single-center quality improvement (QI) initiative at the Miami VA Hospital, a 367-bed inpatient facility that provides primary and specialty healthcare services to over 50,000 veterans. Adults admitted between August 2022 and April 2023 were screened for PCN allergy through an internal report created by the hospital’s health informatics team and reviewed daily by the ASP pharmacist. In-depth pharmacy and allergy histories were reviewed in the electronic health record (EHR), and a bedside patient interview was performed (Figure 1). PCN allergy history was risk-stratified, and the recommended plan was determined by applying this information into a locally developed algorithm (Figure 2). Eligible patients were not offered ADC if they were medically unstable, unable to provide informed consent, or enrolled in hospice. The ASP pharmacist was allotted 3 hours weekly of protected time for this initiative. Work was performed weekdays during normal business hours.

ADC was performed at the bedside as a single dose of amoxicillin 500 mg orally followed by 1 hour of observation by the ASP pharmacist. Informed consent was obtained from all patients. In case of allergic reaction, the hospital’s standard operating procedure for inpatient anaphylaxis was used, which allows the nurse to give the first dose of epinephrine and call the Rapid Response team. Patients were contacted the next day to determine if delayed type 1 hypersensitivity reaction had occurred. Patients were advised to contact the ASP pharmacist if a new rash developed within the following month. Results were documented in the EHR, and the PCN allergy was removed if patient tolerated ADC without symptoms. Written information about PCN allergy clearance was provided to all delabeled patients to decrease the risk of future incorrect relabeling.

### Data collection and outcome measures

Data were collected as part of a QI initiative approved by the facility’s institutional review board. Data collected included patient age, sex, PCN reaction history, risk stratification, number of ADCs, reactions to ADC, and number of patients delabeled. Descriptive analytics was performed on Microsoft Excel.

## Results

There were 1,858 unique patients admitted to a medical or surgical service during the project period. One hundred twenty-two unique patients (7%) were labeled as PCN-allergic. Of these, PCN allergy evaluations were performed on 93 patients (76%). The other 29 patients were missed opportunities due to weekend admissions or rapid discharge planning. Most patients were male (*n* = 83, 78%) with an average age of 69 years (median 71, IQR 15). The most common reaction histories were unknown reaction (*n* = 38, 41%) and rash (*n* = 30, 32%). Most reactions had occurred >10 years prior (*n* = 82, 89%).

Sixty-eight patients (73%) had a low-risk PCN allergy history. Of these, 13 patients were delabeled by history alone and 55 patients qualified for ADC. The most common reason for delabeling based on history alone was documented tolerance of a PCN-class antibiotic since time of reaction (12 patients, 92%). ADC was not offered to 24 patients due to unstable medical condition (18 patients) or mental status (6 patients). Eight patients declined ADC. Eight patients were discharged prior to being challenged. ADC was performed on the remaining 15 patients. There were no immediate type 1 hypersensitivity reactions. One patient developed a mild maculopapular rash after 30 days that did not require treatment (categorized as delayed reaction out of precaution since no other cause could be clearly identified). Twenty-five patients (27%) had high-risk PCN allergy histories. One high-risk patient was delabeled by history due to a well-documented tolerance to PCN-class antibiotics since the time of reaction. In total, 28 patients (30%) were delabeled, including 14 by history and 14 by successful ADC.

## Discussion

There is increasing interest in training nonallergists to assist with PCN allergy delabeling.^5,7,10^ By targeting patients with low-risk PCN allergy histories, an ASP pharmacist was able to implement a delabeling programme at our hospital that worked independently from the Allergy/Immunology service. Due to this autonomy, we used a more conservative risk stratification algorithm compared to other published PCN allergy scoring tools.^6^ Specifically, we used a longer reaction timing (>10 years) and required well-documented evidence of repeat ingestion and tolerance of a PCN-class antibiotic in patients with more recent reactions. Despite this more conservative approach, we, like others, found that most patients were low risk.^6,8,9^, ADC was well tolerated, and there were no type 1 hypersensitivity reactions. All patients maintained a stable level of care without delays in discharge. A limitation of our programme is that we did not perform follow-up after discharge to confirm the absence of delayed-type hypersensitivity.

There were several barriers to performing ADCs on inpatients. Forty-three percent of eligible patients could not be challenged due to an acute medical condition or unstable mental status. Other missed opportunities included rapid discharges and weekend admissions. To recover some of these patients, a pharmacist clinic has been established to offer outpatient ADC after discharge. Implementation of this workflow using a “Train the Trainer” model may expand access to PCN allergy delabeling in healthcare facilities who have limited access to an Allergy/Immunology service.
